# Vital bleaching for children with dental anomalies: EAPD members’ survey

**DOI:** 10.1007/s40368-019-00494-w

**Published:** 2019-11-29

**Authors:** J. Monteiro, P. F. Ashley, S. Parekh

**Affiliations:** Eastman Dental Institute, London, UK

**Keywords:** Bleaching, Whitening, Dental anomalies, Child, Paediatric, Discolouration

## Abstract

**Aim:**

Understand EAPD members’ practices of vital bleaching for children with dental anomalies.

**Methods:**

An anonymous online survey sent via EAPD in January 2019, consisting of 13 questions with possible multiple answers and free text.

**Results:**

110 responses from 24 countries were obtained. The majority worked in hospitals/universities (*n *= 69, 63%) or private practices (*n *= 50, 46%) and were specialists (*n *= 62, 57%) or senior academics (*n *= 35, 32%). Most respondents (*n *= 74 68%) did not provide vital bleaching for children. 88 respondents (80%) belonged to EU: of these, 46 (52%) were not aware of bleaching regulations. For respondents who provided bleaching 26 (72%) undertook home bleaching, using 10% carbamide peroxide (*n *= 21, 58%), most commonly for 2 weeks (*n *= 14, 39%), following establishment of the permanent dentition (*n *= 21, 58%). Deciding factors included: extent (*n *= 27, 75%) and shade (*n *= 26, 72%) of discolouration and child being teased by peers (*n *= 23, 64%). Main reasons for not bleaching included: concerns with side effects (*n *= 41; 55%) and not agreeing with bleaching (*n *= 23, 31%). Dentists who did not bleach managed a range of conditions, most frequently molar-incisor hypomineralisation (*n *= 57; 77%). The majority provided composite restorations with removal of tooth structure (*n *= 50; 68%) with a number opting for no treatment (*n *= 27, 37%).

**Conclusion:**

This study shows wide variations in treatment of children’s dental anomalies across Europe. Fears of adverse effects and personal beliefs seemed to be the main deterrents to bleaching in children. Clinicians who provided bleaching tended to opt for more conservative techniques and to take children’s concerns into consideration.

## Introduction

Intrinsic tooth discoloration in children is commonly associated with multifactorial disorders such as molar-incisor hypomineralisation (MIH), genetic defects including amelogenesis imperfecta (AI) or acquired defects such as fluorosis.

Aesthetic management is important as children as young as 4-year-old report being teased by peers and feeling psychologically affected by tooth discolouration (Rodd et al. 2011; Soares et al. 2015). Conservative/minimally invasive options should be used to maintain enough tooth structure for future restorative treatment and to reduce the burden of care for children who may be anxious and who are likely to face a lifetime of treatment due to their dental disease (Lundgren et al. 2018).

A wide range of therapeutic approaches has been reported, including bleaching, microabrasion, resin infiltration, direct or indirect composite restorations, ceramic veneers and crowns. However, difficulties in tolerating treatment, bonding issues and immature gingival margins pose significant challenges in providing care for these children.

Minimal intervention, whenever possible, is preferred in children, as this is more easily accepted and leads to greater quality of life (Lundgren et al. 2015; Hasmun et al. 2018). Vital bleaching is a minimally invasive treatment, with few side effects, that is effective in improving aesthetics for most patients with enamel and dentine discolouration (Nathwani and Kelleher 2010; Bidra and Uribe 2011; Di Giovanni et al. 2018; da Cunha Coelho et al. 2019).

The American Academy of Paediatric Dentistry developed guidelines for clinicians providing bleaching for children and this technique is freely available in the US (AAPD 2019). In countries belonging to the European Union, directive 2011/84/EU regulates provision of bleaching agents, with a 2012 amendment allowing for up to 6% peroxide hydroxide release in adults. In children, however, restrictions are greater, as tooth whitening agents containing or releasing more than 0.1% Hydrogen Peroxide are not to be used in patients under the age of 18 years. This concentration, however, is not clinically effective, rendering bleaching for children impossible under current regulations (Kelleher 2014). This poses an ethical predicament to clinicians who are, by law, prevented from providing this treatment, with the knowledge that alternatives are often more invasive.

To gain knowledge and understanding of European paediatric dentists’ treatment decisions, opinions and barriers to bleaching, the authors conducted an EAPD members’ survey of current practices of bleaching for children with dental anomalies.

## Materials and methods

An anonymous questionnaire was sent to all members of the European Academy of Paediatric Dentistry (EAPD) using an online survey tool (sogosurvey^®^). The questionnaire was active for 1 month, following an email from the EAPD secretary to its members in February 2019. The email contained an invite for participation, brief instructions and a link to the web-based survey, which was discontinued in March 2019. At this time the number of EAPD members was estimated at over 600.

**Fig. 1:**
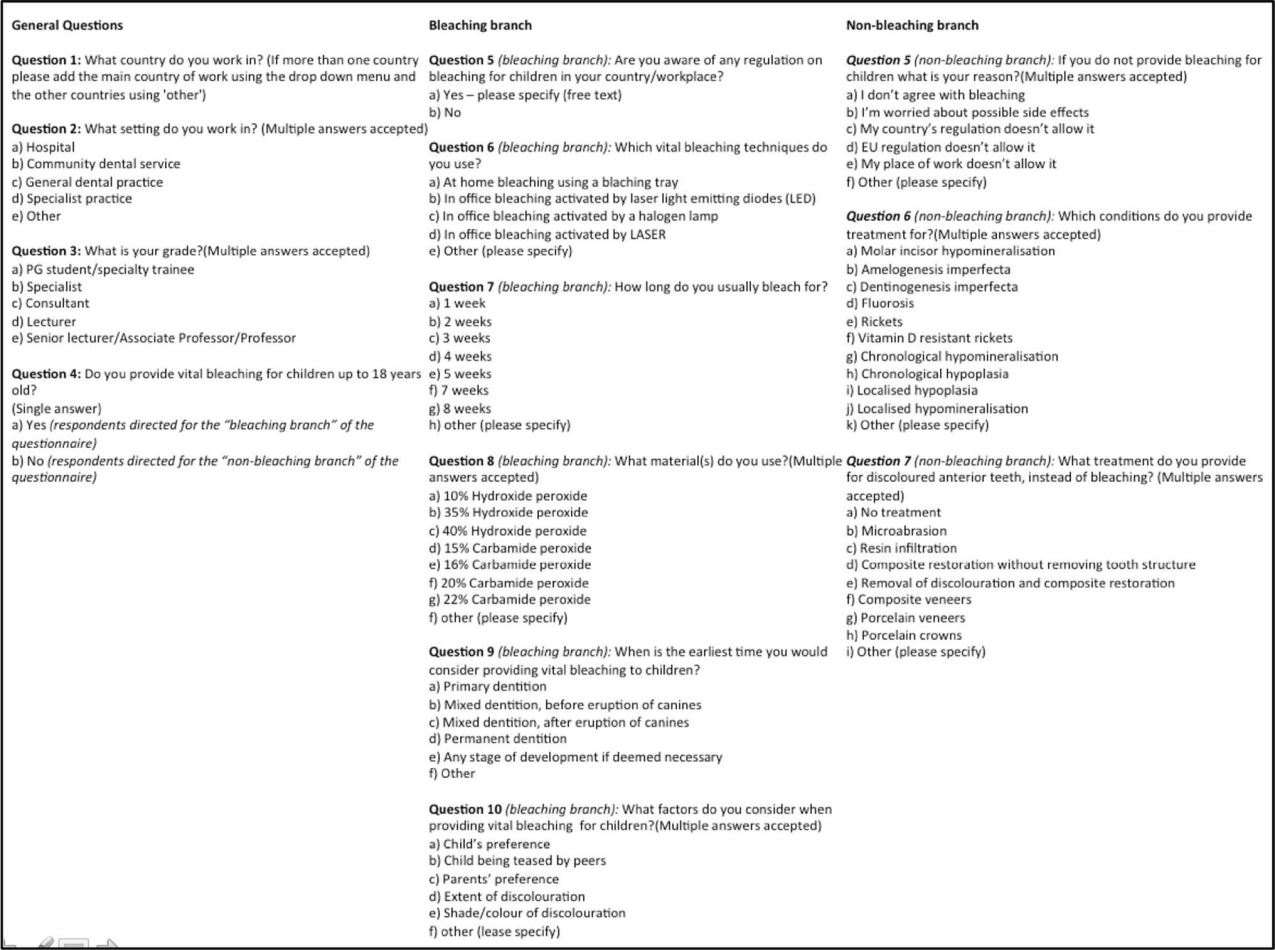
Questionnaire distributed to EAPD members via an online survey provider

The survey consisted of 13 questions relating to vital bleaching for children with dental anomalies. It included multiple-choice questions, with the possibility to select ‘other’ and add free text. Questions were developed having in mind the most frequently occurring dental anomalies in children, current bleaching practices and barriers or concerns as reported in the literature (Kelleher 2014). The number of selected options was unlimited and all answers were included in the analysis. Paediatric dentists from three European countries piloted the questionnaire and comments were incorporated as changes in the final survey (Fig. 1).

Firstly respondents were asked about their country of practice, place of work and professional grade. Following this, respondents were asked whether they provided bleaching for children and their knowledge of any workplace/country bleaching regulations was explored. Depending on this answer, participants were directed to different branches of the questionnaire: Respondents who replied affirmatively were asked about techniques and deciding factors. Respondents who said that they did not bleach were asked about the reasons for this decision, which dental anomalies they treated and which treatment was provided.

## Results

110 respondents from 24 different European countries completed the survey. Israel is a member of the EAPD and for that reason was included in this paper. Non-EAPD member countries are excluded from this paper.

### Work place and professional grade

The majority of respondents worked in hospitals or universities (69 respondents; 63%) and/or in specialist practices (*n *= 50; 46%). Nine (8%) respondents worked in general practice and 7 (6%) worked in community dental services.

**Fig. 2:**
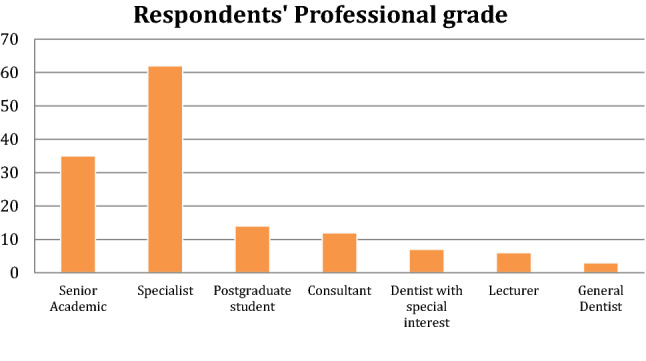
Professional grade of the survey respondents

When asked about their professional grades the majority were specialists (62 respondents, 57%) or senior academics (35 respondents, 32%) (Fig. 2).

### Provision of vital bleaching for children and knowledge of local regulations

The majority of dentists (*n *= 74, 68%) did not provide vital bleaching for children and 62 (56%) were not aware of any regulations in their countries or workplace (Table 1). 88 respondents belonged to EU countries (see EU countries highlighted in grey in Table 1). Of these, over half the respondents (*n *= 46; 52%) were not aware of any regulation on bleaching for children. The majority of those who were bleaching were from the UK and most were aware of regulations in their country.

**Table 1:** Responses according to country (EU countries in italics)

Country	Number of respondents	Number of respondents that bleached	Number of respondents aware of bleaching regulations in their country/workplace
*Austria*	*4*	*1*	*0*
*Belgium*	*7*	*0*	*5*
Bosnia and Herzegovina	1	0	0
*Croatia*	*1*	*0*	*0*
*Cyprus*	*3*	*1*	*0*
*Czech Republic*	*3*	*0*	*1*
*Denmark*	*1*	*1*	*1*
*France*	*2*	*0*	*2*
*Germany*	*3*	*0*	*0*
*Greece*	*21*	*5*	*3*
*Ireland*	*2*	*1*	*2*
Israel	3	0	0
*Italy*	*1*	*0*	*0*
*Netherlands*	*8*	*0*	*3*
Norway	5	2	5
*Portugal*	*4*	*0*	*4*
*Romania*	*2*	*0*	*0*
Slovenia	1	0	0
*Spain*	*3*	*2*	*1*
*Sweden*	*2*	*1*	*2*
Switzerland	3	0	0
Turkey	7	3	1
Ukraine	2	0	0
*United Kingdom*	*21*	*19*	*18*
*Total*	*110*	*36 (33%)*	*48 (44%)*

### Bleaching techniques and deciding factors

The majority of respondents (*n *= 26; 72%) provided at home bleaching, using a bleaching tray. In Office, LED and LASER were rarely used by respondents.

**Fig. 3:**
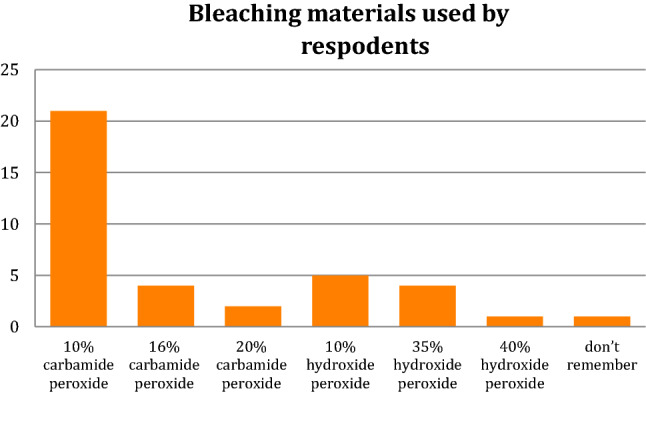
Bleaching materials used by respondents

Regarding bleaching materials, 21 (58%) respondents use 10% carbamide peroxide, with other materials being used less frequently (Fig. 3).

When asked about the earliest time they may consider vital bleaching for children and length of bleaching, the majority stated they would do so in the permanent dentition (*n *= 21; 58.3%). 14 (39%) respondents bleached for 2 weeks. 11 respondents selected ‘other’ to add additional freetext as follows: bleaching duration depends on severity of the discolouration and on the child’s opinion, one respondent discussed that several months may be needed for tetracycline staining. The most frequent deciding factors for vital bleaching in children were: extent of discolouration (*n *= 27; 75%), shade/colour of the discolouration (*n *= 26; 72%), child being teased by peers (*n *= 23, 64%) (Table 2). Other factors added to free text included psychosocial effects on children, type of discolouration, compliance and whether the clinician thought that vital bleaching would resolve the discolouration.

**Table 2:** Bleaching deciding factors, earliest time bleaching was considered and treatment length

Deciding factors for bleaching	
Extent of discolouration	*n *= 27 (75%)
Shade/colour of the discolouration	*n *= 26 (72%)
Child being teased by peers	*n *= 23 (64%)
Child’s preference	*n *= 13 (36%)
Parents’ preference	*n *= 8 (22%)

When is the earliest time you would consider providing vital bleaching to children?	
Primary dentition	*n *= 1 (2.7%)
Mixed dentition, before eruption of canines	*n *= 2 (5.5%)
Mixed dentition, after eruption of canines	*n *= 7 (19%)
Permanent dentition	*n *= 21 (58%)
Any stage of development, if deemed necessary	*n *= 5 (13%)

How long do you usually bleach for?	
1 week	*n *= 4 (11%)
2 weeks	*n *= 14 (39%)
4 weeks	*n *= 6 (17%)
6 weeks	*n *= 1 (2.7%)

23 respondents (21 belonging to EU countries), mainly working in hospitals or universities, said they were aware of bleaching regulations, but they would provide bleaching to their child patients. There were similar numbers of PG students/trainees, specialists, senior lecturers and consultants. All performed at home bleaching using a tray, mostly in the permanent or mixed dentition after the canines had erupted and they had a variety of reasons for bleaching (Table 3). In free text some respondents added: “multifactorial reasons for bleaching, as alternative to more invasive procedures”, “sometimes bleaching does the trick so the child is satisfied and we can postpone any prosthodontics therapy till they are older”.

**Table 3:** Responses from dentists who provided bleaching, being aware of bleaching regulations

Country	Number of respondents	Regulation	Justification for bleaching
UK	18	EU directive (14) Free text: “Local approval” (1) Free text: “Individual defence societies” (1) Free text: “Royal College of surgeons” (1) Free text: “Cannot name” (1)	Child being teased by peers (*n *= 17) Extent of discolouration (*n *= 16) Shade/colour of discolouration (*n *= 14) Child’s preference (*n *= 10) Parents’ preference (*n *= 4) Free text: “Negative psychosocial factors” “Clinical indication after assessment of other options” “Evidence of discolouration actually causing psychological harm to the child” “Compliance” “If I think bleaching would help resolve the issue”
Norway	2	EU directive (*n *= 1) Free text: “Not recommended under 18” (*n *= 1)	Multifactorial (*n *= 1) Extent of discolouration (*n *= 1) Shade/colour of discolouration (*n *= 1) Free text: “Child must consider it is a problem to him/her” “The child must be very cooperative and mature”
Ireland	1	EU directive	Child being teased by peers Extent of discolouration Shade/colour of discolouration Child’s preference Parents’ preference
Spain	1	Free text: “Not allowed under 18”	Extent of discolouration Shade/colour of discolouration
Sweden	1	EU directive	Free text: “When there is an odontological indication like fluorosis or AI”

### Reasons for not providing vital bleaching to children and alternative treatments for children with dental anomalies

**Fig. 4:**
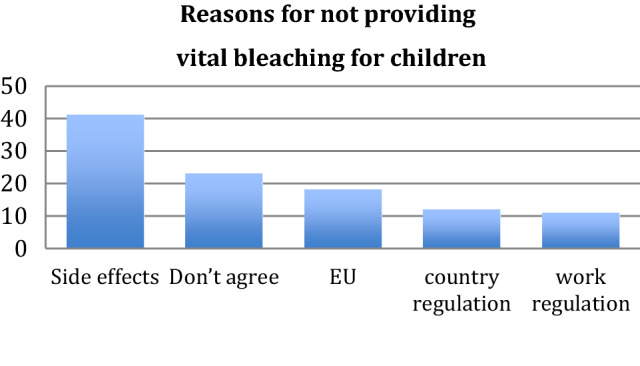
Respondents’ reasons for not providing vital bleaching to children

Respondents who did not provide vital bleaching for children were worried about possible side effects (*n *= 41; 55%) or regulations (*n *= 41; 55%), with only 18 (24%) mentioning EU regulations as an impediment (Fig. 4). Other reasons given as free text included: “there is no indication for bleaching”, “there are other priorities in hospital dentistry”, “it is not important for paediatric dentistry”, it is “too early”, “must be delayed until the age of 18” and one respondent reported “no experience in bleaching”.

There were a variety of conditions treated by this group of clinicians, most frequently, molar-incisor hypomineralisation (*n *= 57; 77%). When asked about which treatment would they provide for discoloured anterior teeth instead for bleaching, the majority responded removal of discolouration and composite restoration (*n *= 50; 68%) (Table 4).

**Table 4:** Dental anomalies treated by respondents who did not bleach and treatment provided—although hypoplasia may not be associated with intrinsic discolouration it can be associated with secondary extrinsic discolouration of rough or pitted surfaces

Conditions treated by respondents who do not bleach	
Molar-incisor hypomineralisation (MIH)	*n *= 57 (77%)
Localised hypomineralisation	*n *= 47 (64%)
Fluorosis	*n *= 40 (54%)
Amelogenesis imperfecta	*n *= 40 (54%)
Localised hypoplasia	*n *= 40 (54%)
Chronological hypomineralisation	*n *= 32 (43%)
Dentinogenesis imperfecta	*n *= 29 (39%)
Chronological hypoplasia	*n *= 26 (35%)
Vitamin D resistant rickets	*n *= 13 (17%)
Rickets	*n *= 11 (15%)
Tetracycline staining (free text)	*n *= 1 (1.4%)

Treatment provided to discoloured anterior teeth instead of vital bleaching	
No treatment	*n *= 27 (37%)
Microabrasion	*n *= 41 (55%)
Resin infiltration	*n *= 37 (50%)
Composite restoration without removing tooth structure	*n *= 34 (46%)
Removal of discolouration and composite restoration	*n *= 50 (68%)
Composite veneers	*n *= 20 (27%)
Porcelain veneers	*n *= 5 (6.8%)
Porcelain crowns	*n *= 2 (2.7%)

## Discussion

This survey had a low response rate with only 110 out of over 600 EAPD members participating in this survey. A possible language barrier and our decision to exclude non-EAPD member countries in this article may explain this response rate.

There was some confusion regarding the EU directive: 88 respondents worked in EU countries, but only 52% of these were aware of any bleaching regulations, and a smaller percentage referred to Directive 2011/84/EU. In fact, fear of adverse reactions and personal beliefs were the major deterrents to bleaching, not the EU directive. It is important to understand that the most commonly occurring side effects of bleaching are transient sensitivity and gingival irritation (Donly et al. 2005). Increased sensitivity and poor oral hygiene associated with certain enamel defects may deter some clinicians from bleaching. Nevertheless, thorough patient instructions, well-fitting bleaching trays, reduced exposure to bleaching products and prescription of desensitizing agents may help address this issue. Although common, these are temporary adverse reactions that resolve following discontinuation of treatment (Donly et al. 2005; Donly 2010; Greenwall-Cohen et al. 2018).

Clinicians that did not bleach opted for less conservative treatment approaches. The majority of these options involved removal of tooth structure to facilitate masking of opacities. Although this may be necessary in a small number of cases, minimally invasive techniques should be the treatment of choice, whenever possible.

Some respondents discussed that they would not provide any treatment for children less than 18 years of age. This may be a valid approach for children who have no pain or psychosocial complaints from their aesthetic challenges. The consequences of discolouration, however, pose a significant impact on children’s psychosocial well-being, with studies reporting they may feel self-conscious, have low self-esteem and avoid smiling or socializing (Marshman et al. 2009; Parekh et al. 2014). Children as young as pre-school and primary school ages are already aware of their own noticeable differences and can judge affected peers negatively (Rodd et al. 2011; Craig et al. 2015; Soares et al. 2015; Lundgren et al. 2016). For this reason, parents report being worried about bullying, and tend to seek treatment early, especially prior to milestones such as starting school (Alqadi and O’Connell 2018).

Unfortunately young adults with AI have discussed they felt that their opinions were often not considered and consequently felt they were not involved in their own treatment decisions as children (Lundgren et al. 2016). In this survey, clinicians providing bleaching reported that the main deciding factors for this treatment were clinical presentation and the concerns of children and their parents. Indeed this seemed to be the driver for respondents to provide bleaching treatment to children with anomalies, with a number of clinicians opting to bleach despite knowledge of regulations. These clinicians considered severity of disease, the child being bullied by peers and the alternative of more destructive treatments in their decision-making. Additionally, respondents that replied affirmatively to bleaching tended to adopt conservative bleaching techniques such as using low concentrations of carbamide peroxide (10%), at home bleaching, bleaching in the adult or mixed dentitions only and either limiting to 2 weeks or stopping when the child was happy.

A large number of respondents were aware of the regulation and decided to continue bleaching if they felt this was the best treatment option for their patients. This seems to be the case predominantly in the UK, where the General Dental Council has stated that bleaching would be accepted for treatment of disease. Nevertheless controversy remains as EU law is still in place in the UK.

## Conclusion

This survey found a wide variation in treatment of dental anomalies in European children. Although bleaching is one of the most conservative options, fears of adverse effects and dentists’ personal beliefs seem to be major deterrents to providing this treatment to children. Clinicians who undertake dental bleaching often opt for more conservative bleaching techniques and take clinical presentation as well as children’s views into consideration.

## References

[CR1] AAPD. American Academy of Paediatric Dentistry, policy on the use of dental bleaching for child and adolescent patients, latest revision. 2019. https://www.aapd.org/media/Policies_Guidelines/P_Bleaching.pdf. Accessed Sep 2019.

[CR2] Alqadi A, O’Connell AC (2018). Parental perception of children affected by amelogenesis imperfecta (AI) and dentinogenesis imperfecta (DI): a qualitative study. Dent J (Basel)..

[CR3] Bidra AS, Uribe F (2011). Successful bleaching of teeth with dentinogenesis imperfecta discoloration: a case report. J Esthet Restor Dent..

[CR4] Craig SA, Baker SR, Rodd HD (2015). How do children view other children who have visible enamel defects?. Int J Paediatr Dent.

[CR5] Da Cunha Coelho ASE, Mata PCM, Lino CA (2019). Dental hypomineralization treatment: a systematic review. J Esthet Restor Dent.

[CR6] Di Giovanni T, Eliades T, Papageorgiou SN (2018). Interventions for dental fluorosis: a systematic review. J Esthet Restor Dent.

[CR7] Donly KJ, Kennedy P, Segura A, Gerlach RW (2005). Effectiveness and safety of tooth bleaching in teenagers. Paediatr Dent.

[CR8] Donly KJ (2010). A controlled clinical trial to evaluate the safety and bleaching efficacy of a 9.5% hydrogen peroxide high-adhesion bleaching strip in a teen population. Am J Dent.

[CR9] Greenwall-Cohen J, Greenwall L, Haywood V, Harley K (2018). Tooth whitening for the under-18-year-old patient. Br Dent J.

[CR10] Hasmun N, Lawson J, Vettore MV (2018). Change in oral health-related quality of life following minimally invasive aesthetic treatment for children with molar incisor hypomineralisation: a prospective study. Dent J (Basel).

[CR11] Kelleher M (2014). The law is an ass: legal and ethical issues surrounding the bleaching of young patients’ discoloured teeth. FDJ.

[CR12] Lundgren PG, Karsten A, Dahllöf G (2015). Oral health-related quality of life before and after crown therapy in young patients with amelogenesis imperfecta. Health Qual Life Outcomes.

[CR13] Lundgren PG, Wickström A, Hasselblad T, Dahllöf G (2016). Amelogenesis imperfecta and early restorative crown therapy: an interview study with adolescents and young adults on their experiences. PLoS One.

[CR14] Lundgren GP, Vestlund GM, Dahllöf G (2018). Crown therapy in young individuals with amelogenesis imperfecta: long term follow-up of a randomized controlled trial. J Dent.

[CR15] Lundgren PG, Hasselblad T, Johansson AS, Dahllöf G (2019). Experiences of being a parent to a child with amelogenesis imperfecta. Dent J.

[CR16] Marshman Z, Gibson B, Robinson Z, Gibson B, Robinson PG (2009). The impact of developmental defects of enamel on young people in the UK. Commun Dent Oral Epidemiol.

[CR17] Nathwani NS, Kelleher M (2010). Minimally destructive management of amelogenesis imperfecta and hypodontia with bleaching and bonding. Dent Update..

[CR18] Parekh S, Almehateb M, Cunningham SJ (2014). How do children with amelogenesis imperfecta feel about their teeth?. Int J Paediatr Dent.

[CR19] Rodd HD, Marshman Z, Gibson B (2011). Oral health-related quality of life of children in relation to dental appearance and educational transition. Br Dent J.

[CR20] Soares FC, Cardoso M, Bolan M (2015). Altered esthetics in primary central incisors: the child’s perception. Pediatr Dent.

